# Molecular investigation of a RSV outbreak in a geriatric hospital

**DOI:** 10.1186/s12877-021-02064-6

**Published:** 2021-02-12

**Authors:** Yohan Hababou, Assia Taleb, Amélie Recoing, Frédérique Moreau, Isabelle Simon, Florence Muller de Schongor, Elyanne Gault, Marie-Anne Rameix-Welti

**Affiliations:** 1 AP-HP, Université Paris Saclay, Hôpital Ambroise Paré, Laboratoire de Microbiologie, Boulogne-Billancourt, France; 2 AP-HP, Université Paris Saclay, Hôpital Sainte Perrine, Equipe opérationnelle d’hygiène, Paris, France; 3 AP-HP, Université Paris Saclay, Hôpital Sainte Perrine, Service de gériatrie, Paris, France; 4 Université Paris-Saclay, INSERM, Université de Versailles St. Quentin, UMR 1173 (2I), Versailles, France

## Abstract

**Background:**

Acquired infections in hospitalized elderly people are a growing concern. In long-term care facilities with multiple staff and visitor contacts, virus outbreaks are a common challenge for infection prevention teams. Although several studies have reported nosocomial RSV outbreaks in long term care facilities, molecular epidemiology data are scarce.

**Methods:**

RSV RNA was detected in respiratory samples from 19 patients in a long-term care hospital for elderly in Paris in March 2019 over a 3 weeks period. Genotyping was performed using nucleotide sequencing. Sociodemographic and clinical characteristics of cases part of a unique cluster, were retrospectively reviewed.

**Results:**

Molecular investigation of theses RSV cases, revealed a unique cluster of 12 nosocomial cases in 2 adjacent wards. Mean age of these outbreak’s cases was 89. All patients had underlying medical conditions. Seven exhibited lower respiratory symptoms and three experienced decompensation of underlying chronic heart condition. Two patients died.

**Conclusions:**

This case report highlights the importance of RSV in causing substantial disease in elderly in case of nosocomial outbreak and the contributions of molecular epidemiology in investigation and management of such outbreak.

**Supplementary Information:**

The online version contains supplementary material available at 10.1186/s12877-021-02064-6.

## Background

Population ageing is a global phenomenon with increase of both the size and the proportion of older persons in the population. European countries currently have the greatest percentage of older population (25%). In 2019, there were 703 million persons over 65 years of age worldwide and this number is projected to double in 2050 [1]. Elderly people are especially vulnerable to infections, including respiratory infections. More than 85% of all deaths from lower respiratory tract infections occur in adults over 70 years in high income countries [2]. The severity of respiratory infections in elderly was particularly highlighted during the recent COVID-19 pandemic [3]. Contributing factors for increased susceptibility to infection and poor health outcomes include immunosenescence, age related decay of physiological functions and high frequency of comorbid conditions. Decrease of the forced expiratory volume in 1 s / forced vital capacity (FEV_1_/FVC) with age in the absence of any pathological conditions is an illustration of age related decline of lung function [4]. A large population study in UK reported that 64.9% of adults between 65 and 84 years old and more than 80% of adults over 85 years old exhibited at least two chronic conditions [5]. The number of long-term care facilities for the elderly (LTCF) have increased in modern countries. For instance in France, 10% of people over 70 years old and one third of people over 90 years old are living LCFT in France [6]. The long-term hospitalization geriatric unities, characterized with multiple staff and visitors contact and altered mental status patient, are facing with the burden of nosocomial infection. Understanding the origin and transmission dynamics of the outbreak is needed to design efficient measures to prevent virus spread.

RSV belongs to the family of *pneumoviridae*. The variability of the surface glycoprotein G, in particular of its hypervariable region, is at the origin of the classification of RSV in multiple genotypes divided into 2 groups (A and B) [7]. RSV is the leading cause of lower respiratory tract infections in newborn children worldwide, and the main etiological agent of infant bronchiolitis. RSV accounts for 33.8 million bronchiolitis cases each year, leading to the hospitalization of more than 3 million children under 5 years of age worldwide [8]. It is now recognized as a growing cause of respiratory disease in the elderly, particularly among those immunosuppressed or with underlying conditions such as chronic respiratory or cardiac disease. Indeed, RSV is detected in approximately 10% respiratory illnesses in hospitalized elderly and is associated with a case fatality rate around 10% [9–12]. To date, no curative antiviral drugs or vaccines have been commercialized. Nosocomial RSV outbreaks have been reported mostly in neonatal intensive care and hematology units [13–16] but also in LTCF for elderly [17–20]. Here, we describe a nosocomial RSV outbreak that occurred in March 2019 in a French long-term stay hospital for elderly and illustrate the benefit of molecular investigations in the understanding and control of virus spread.

## Methods

### Outbreak settings

Sainte-Perine-Chardon-Lagache-Rossini hospital (SPR-CLR) is a 598 beds geriatric academic hospital in Paris, France. This hospital is part of the “Paris Ile-de-France Ouest” hospital group (GHUPIFO) which also encompasses two general academic hospitals, Ambroise Paré (APR) in Boulogne-Billancourt and Raymond Poincaré in Garches (859 beds including general and intensive care pediatric units and pediatric and adult emergency units).

SPR-CLR consists of three separate buildings: SPR, CLR and ROS, that operate with independent staff. SPR building has 298 beds distributed in 8 wards on 2 floors. SPR-R ward is in the ground floor and consists in 11 double and 15 single occupancy rooms. SPR-M and SPR-C wards are in the first floor. SPR-M ward consists in 11 double and 15 single occupancy rooms. The stairs or the lift can be used by visitors, medical staff and patients to access to the different wards of the SPR building. In this building, mean patient age is 89 years, and patients suffer from related-age disease such as dementia. Some of them can ambulate, independently or with help.

Patients from SPR can meet each other in a common dining room and in a physical therapy room located in the ground floor. Furthermore, patients and visitors can relax in a park in the SPR building entrance.

### Samples and diagnostics

Respiratory specimens (nasal swabs) were collected from patients as part of routine care. No additional samples were collected in the course of this work. Swabs were screened for respiratory pathogenic agents (adenovirus, coronavirus (HKU1, NL63, 229 E, OC43), influenza A(H3N2), A(H1N1) and B viruses, human metapneumovirus, parainfluenza 1 to 4 viruses, respiratory syncytial virus, rhinovirus/enterovirus, chlamydia pneumoniae, mycoplasma pneumoniae, bordetella pertussis and parapertussis) using multiplex reverse transcriptase (Biofire® Filmarray® technology) in the GHUPIFO virology lab. Samples were processed within 24 h, and then stored at − 80 °C.

### RSV genotyping

Genotyping was performed on all respiratory specimens tested positive for RSV in the GHUPIFO virology lab throughout March 2019. Total nucleic acids were extracted from the remaining respiratory specimens stored at − 80 °C using the MagNA Pure Compact technology (MagNA Pure Compact Nucleic Acid Isolation Kit, Roche®) according to the manufacturer’s protocol. Amplification of the second hypervariable region of the G gene of RSV-A and RSV-B were performed with One Step RNA PCR kit (Takara®) using specific primers (RSV-A 5′-GAAGTGTTCAATTTTGTACC and 5′-GATTGGCAACTCCATTGTTATTTGC; RSV-B 5′-GAAGTGTTCAACTTCGTTCC and 5′-GATCAGCAACTCCATGGTTATTTGC). After size verification (around 500 bp), the amplified DNA was sequenced with BigDye® Terminator Cycle Sequencing Kit (Applied Bioystems) using 3500xL Dx Genetic Analyzer HITACHI®. Sequences were aligned on RSV-A and RSV-B references (JF920052 and KU950542) using CLC main Workbench® software. Genotype was assigned by phylogenetic clustering against the GenBank reference sequences set from Gaymard et al [21] by neighbor-joining method. For the B group phylogeny, the number of BA sequence was limited to 14 representative ones.

### Infection control interventions

Droplets and contact precautions were implemented for patients with possible or confirmed viral infection. These precautions consist in i) confinement of the patient in one’s room until fully recovered. If a single bed-room is not available, the neighbor patient must wear a surgical mask. ii) Use of personal protection equipment for the staff: medical mask, gloves when in touch with patients or potential infected surfaces and hand-washing by hydro-alcoholic solutions when the care is over. iii) Temporally suspension of common activities (including common lunch room attending).

The outbreak infection control plan also included the following precautions to prevent RSV diffusion:

- Reminder on respiratory hygiene (covering the mouth when coughing, use disposable tissue) and on hand hygiene (use of hydro-alcoholic solutions) for patients and staff.
- Visitor restriction. For authorized visitors, use of respiratory mask and extensive hand disinfection.
- Surface disinfection twice a day of areas possibly touched by residents (door arms, switch, bed rails, phone…). Use of disposable devices when possible, otherwise use of dedicated material daily disinfected.
- Proper ventilation of the rooms e.g. six times air renewing per day.

### Review of the medical records and ethics

Demographic, clinical, biological and epidemiological data were collected, retrospectively, from the medical record of every patient with laboratory-confirmed RSV infection during March 2019 in SPR-R and SPR-M wards (considered as “outbreak case”). Data collection, storage and analysis was carried out according to MR004 CNIL standard and declared to the “data protection APHP, Paris Saclay university”. Lack of opposition to participating in clinical research was verified in the records of all patients. Living patients at the time of the study were contacted, informed and did agree to the publication of the data anonymously. Hospital-acquired RSV was defined as RSV confirmed by PCR ≥5 days after admission, based upon the usual incubation period [22]. Symptoms and clinical evolution related to RSV were followed up for 1 month after RSV infection documentation. Probable onset dates were determined with medical record data. The duration of an outbreak was defined as the time (days) elapsed from the onset of the illness in the first proven case to the onset of the illness in the last proven case. The attack rate was calculated as follows:

$$ \frac{Total\  RSV\  laboratory- confirmed\ cases\ in\  SPR-R\  ward\ during\ the\ outbreak\ time\ interval}{ Total\ number\ of\ residents\ in\  SPR-R\  during\ the\ outbreak\ time\ in terval} $$

## Results

**Fig. 1:**
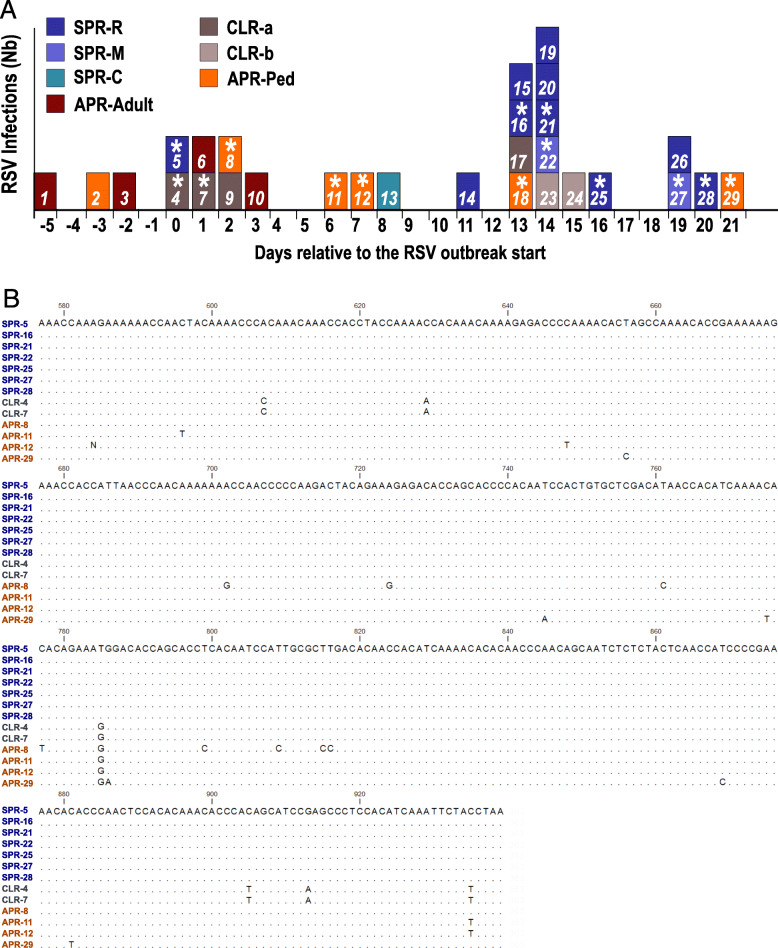
Chronology of the outbreak and molecular analysis. **a**: Distribution of confirmed RSV cases in the hospital group (GHUPIFO) in March in by onset of days relative to the outbreak start. The color identifies the ward involved. SPR-R, −M and -C are 3 adjacent wards in SPR building. Cases from SPR-R and -M (in blue) were considered as the “outbreak cases”. CLRa and b are 2 different wards from CLR building. The “*” symbol indicates successfully sequenced samples. **b**: Sequence alignment of the second variable region of the RSV G gene (nucleotide positions 577 to 939 relative to the start codon). Sequence of RSV-A (ON1) from patient 18 was not included in the alignment. Dots indicate nucleotide identities. RSV G gene nucleotide sequences were deposited in GenBank under accession numbers GenBank MT989450 to MT98945062

Nineteen RSV cases were detected in SPR-CLR geriatric hospital group over a 20-day period in March 2019. This was an unexpectedly high number of RSV cases taking into account that the RSV epidemic was over in Ile-de-France since the end of January 2019 (week 3–4) [14]. By contrast, 15 cases of RSV were detected in SPR-CLR in December 2018 (RSV epidemic peak for Ile-de-France). These 19 RSV infected patients had been hospitalized in SPR-CLR for more than 7 days (mean 56, min 24 max 131 days) when RSV infection occurred, strongly suggesting a nosocomial outbreak. The mean age was 89 ranging from 75 to 99. A chart of all laboratory-confirmed RSV cases detected in the hospital group (GHUPIFO) in March 2019 per day is represented in Fig. 1a. Out of the 19 SPR-CLR cases, 10 were from SPR-R, 2 from SPR-M and one from SPR-C; 6 were from 2 separate wards of CLR building. Apart from the 19 cases in SPR-CLR, we detected 10 other RSV cases in APR general hospital over this time period. Six were pediatric patients and 4 were adults seen in emergency department or hospitalized in acute care units.

To assert the hypothesis of a unique cluster, we performed RSV genotyping based on the sequence of the second hypervariable region of the G gene for the 19 cases in SPR-CLR. The 10 cases from APR were included in this analysis to assess variability of concomitantly circulating RSV in the same geographic area. We were able to establish the genotype of 14 out of the 29 RSV detected in March 2019 (Fig. 1a) (9 out of the 19 RSV from SPR-CLR and 5 out of the 10 control RSV from APR). As the Biofire® Filmarray® relies on highly sensitive nested PCR, failure of genotyping procedure for 15 samples is most likely due to low viral titers in the sample. One RSV-A (ON1) was detected in a pediatric patient (patient 18, Fig. 1a). The others were RSV-B, all belonging to RSV BA genotype. Alignment of their sequences is shown on Fig. 1b.

The 7 analyzed sequences of RSV detected in SPR-R and SPR-M units during this outbreak were exactly identical and exhibited 6 to 9 differences with co-circulating RSV-B isolates analyzed here. These data strongly support the circulation of a unique RSV strain in SPR-R and in SPR-M unit over a 3 weeks period. The 12 RSV cases from SPR-R and SPR-M were thus considered as part the same nosocomial outbreak and are referred as “outbreak cases”. Molecular analysis has also enabled the definition of outbreak start by identifying the source patient (patient 5, Fig. 1a). Strikingly, the 7 studied sequences resulting from person-to-person transmission over 20 days were absolutely identical. On the contrary, the differences observed among specimens of patients 4 and 7, ruled out the hypothesis of an extension of the outbreak in the whole SPR-CLR hospital. Noteworthy, sequences obtained from patients 4 and 7 were identical, suggesting that a small cluster of RSV infections also occurred in a unit of CLR building. Another set of two identical sequences was observed in samples from two children (patients 11 and 12) who were, however, not hospitalized in the same unit.

**Fig. 2:**
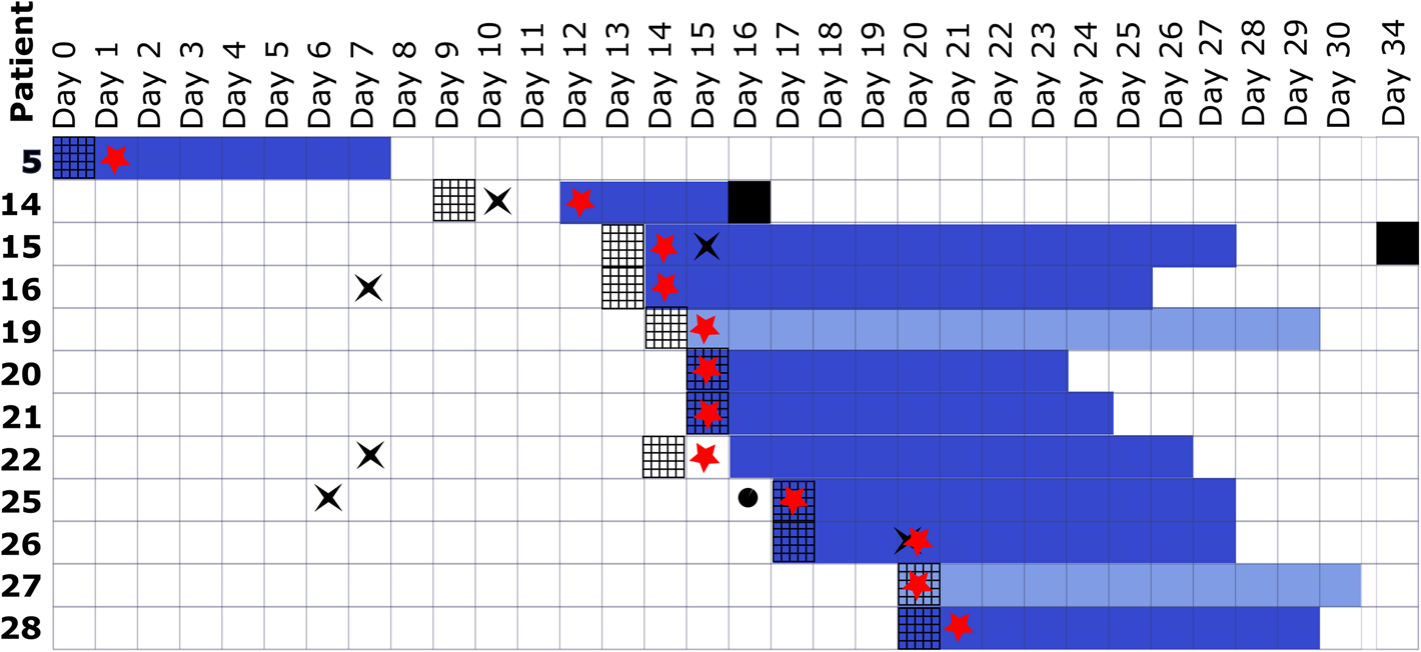
Chronological illustration of clinical and laboratory findings and implementation of droplets precautions for SPR-R and SPR-M RSV patients. Days relative to the start of the outbreak are indicated at the top. Patient numbers are referring to Fig. 1. Solid bars indicate the droplet precaution period (SPR-R dark blue, SPR-M light blue). Black squares indicate patient death. Red stars indicate the laboratory confirmation of RSV infection. Grids indicate the onset of symptoms. Chest X ray (black cross) and physical therapy (black circle) are mentioned

The index case (patient 5) involved a patient transferred from acute geriatric unit to SPR-R long term unit on the 5th of March, who presented with rhinorrhea at admission. Droplet precautions had been implemented at admission and carried on for 7 days, according to the recommended practice. The next outbreak case (patient 14) was detected 9 days later, but the patient was placed into droplets and contact precaution only 3 days after the first respiratory symptoms, because RSV was first misdiagnosed with exacerbation of severe chronic heart failure. Precautions were implemented as soon as the patients were symptomatic for all the following cases (Fig. 2). The fifth first cases were detected in SPR-R ward (ground floor), and then the outbreak reached the SPR-M ward (first floor) from day 14. No other patient was diagnosed after day 20, it can be assumed that the intensified infection control measures stopped further transmission. Unfortunately, staff members were not investigated. Considering only the SPR-R unit and supposing all cases were diagnosed, the attack rate of this outbreak would be 35% (10/28).

The 12 patients’ files were reviewed retrospectively to assess their underlying conditions, symptoms and outcomes. Socio-demographic and clinical data are indicated in the Table 1 and detailed for each case in Supplementary Figure 1. Mean age was 89 years old. All resident cases patients had underlying medical conditions, including undernutrition, mild to severe physical and psychological dependency or chronic heart disease. Three patients had diabetes and two had renal insufficiency on admission are encountered respectively at 3 and 2 patients. Eleven patients out of 12 presented with symptoms. The cough was the most common symptom, encountered in the half of the infected patients, whereas fever only occurred in 3 out of 12 patients. Only 3 patients expressed symptoms limited to upper respiratory tract. By contrast, lower respiratory tract symptoms occurred in 7 patients out of 12 patients. Chest X-ray was performed for 3 patients out of 12 with severity or predictive factors such as high heart rate (> 100), polypnea (> 35) or asymmetrical pulmonary auscultation. Patient 15 exhibited infiltrates on the chest X-ray and was the only patient with CRP over 100 mg/mL (320 mg/mL). Following RSV infection, 3 patients experienced a decompensation of neurological status and 3 patients experienced exacerbation of chronic heart failure (CHF) with pulmonary overload. One of them died due to acute cardiac failure 6 days after RSV infection (patient 2). Patient 15 developed bacterial pneumonia (confirmed by chest X-ray) and died 3 weeks after the RSV outbreak.

**Table 1:** Characteristics of the 12 patients infected by RSV during the outbreak

***Characteristics***		
Age: Median/Mean (Range) (years)	88 / 89 (75–99)	
Men/Women	2 / 10	
***Comorbidities***		
Undernutrition ^a^	3	*(25%)*
Active smoking	1	*(8%)*
Respiratory virus disease over the previous year	1	*(8%)*
Co-infection	1	*(8%)*
Dementia	2	*(17%)*
Chronic Heart Disease	6	*(50%)*
Chronic Respiratory Disease	2	*(17%)*
Diabetes	3	*(25%)*
Active neoplasia or diagnosed over the previous year	2	*(17%)*
***Clinical presentation and outcome***		
Fever ^b^	3	*(25%)*
Neutrophilia ^c^	1	*(8%)*
Serum CRP concentration above 5 mg/L	8	*(67%)*
Acute change in mental status from baseline	3	*(25%)*
URTI ^d^	3	*(25%)*
LRTI ^e^	7	*(58%)*
Cough	6	*(50%)*
Hypoxemia	4	*(33%)*
Decompensation of underlying conditions ^g^	3	*(25%)*
Death	2	*(17%)*
***Treatments***		
Oxygeno therapy ^f^	3	*(25%)*
Antibiotics ^h^	1	*(8%)*
Beta 2 mimetics (aerosol)	5	*(42%)*
Corticosteroids	1	*(8%)*

^a^ Defined by serum albumin concentration below 35 mg/L according to the biological definition by French health authority [23]

^b^ Single oral temperature over 37,8 °C or repeated temperature over 37,2 °C

^c^ Neutrophil count superior to normal (7500/mm^3^)

^d^ Rhino pharyngitis (runny nose, sneezing or stuffy nose) or pharyngitis (sore throat, difficulty in swallowing) or influenza-like illness (fever, chills and myalgia)

^e^ Bronchial obstruction, wheezing or crackles, dyspnea

^f^ Defined by oxygen saturation level below 96%

^g^ Chronic heart failure decompensation

^h^ 7 days antibiotherapy

## Discussion

This study describes the investigation of an RSV outbreak in a long term geriatric hospital. Nosocomial spread of viruses often parallels outbreaks in the community with the possibility of repeated introductions. This can prevent outbreak detection and impede spread monitoring. Molecular investigation enabled us to confirm the epidemiologically suspected outbreak, to pinpoint the source patient and to identify cases. Genotyping indeed linked the cases from two adjacent units while excluding cases from other ones that had been wrongly included. Few previous studies reported molecular investigation of nosocomial RSV outbreaks mostly in neonates and in oncology or onco-hematology wards [13, 18, 20, 24, 25] and demonstrated the usefulness of phylogenetic analysis to include or exclude cases and to identify source patient. For instance, Chu et al. sequenced 15 strains from a nosocomial out break and concluded that only 10 were related, the 5 others corresponding to 3 separate introduction events [26]. Molecular investigation of nosocomial epidemics mainly in onco-hematology departments involving PIV3, influenza viruses and adenoviruses have led to similar conclusions, clearly demonstrating the contribution of sequencing for the identification of related cases [27–29]. Here, sequencing was proved a useful epidemiologic tool to investigate nosocomial outbreak, however only retrospectively. The advent of high-throughput sequencing methods makes it technically possible to quickly obtain molecular information on viral strains [30–32]. Strikingly, recent systematic whole genome analysis of health care associated influenza A, revealed frequent in-ward transmissions leading to small clusters and suggesting under-detection of nosocomial outbreaks [33]. In the future, these approaches could be adapted to real-time monitoring of nosocomial outbreaks, enabling rapid implementation of control measures.

Noteworthy, sequences from RSV isolated over 3 weeks period were exactly identical, as reported in similar studies on RSV or hMPV nosocomial outbreaks, mostly in immunosuppressed patients from onco-hematology units [13, 24, 26, 34]. Our data suggest that RSV stability through transmission is also true in elderly patients.

The calculated attack rate of this outbreak in SPR-R is 35%, in accordance with previously published geriatric outbreak descriptions [20, 35]. RSV outbreaks in long term care geriatric hospital or center are a cause of concern since they result in severe even life-threatening infections [36]. Indeed, reported case fatality rate of such outbreak range between 0 and 14%, and was 17% (2/12 cases) in our study [19, 20, 37]. Control of outbreaks in long term hospitalization geriatric units is thus critical but highly challenging, facing logistic issues related to the congregate setting, residents with dementia, and understaffing. Here, direct patient-to-patient transmission may have occurred since the index patient was independently mobile and exhibited neurological decompensation, making hygiene measures compliance inconstant. Early detection of cases is essential in order to implement the appropriate control measures, but is compromised by atypical presentation of respiratory viral infection in elderly. Indeed, very elderly are likely to express confusion or exacerbation of underlying conditions as only symptoms of respiratory viral infections [38, 39]. In this study, only half of the patients exhibited respiratory symptoms. Two patients exhibited respectively worsening of neurological condition and acute chronic heart failure decompensation as only symptoms of RSV infection. RSV detection was due to systematic investigation in the context of the outbreak. This underlies the need to search for respiratory infection in case of decompensating of underlying chronic condition in very elderly and to implement droplets and contact precautions at the slightest doubt.

Efficacy of cohorting to control an RSV outbreak has been shown in hemato-oncological units [40]. However, in long term hospitalization geriatric units, the lack of free rooms may hinder the implementation of this strategy as reported for an RSV outbreak in dementia care ward [19]. Furthermore, the lack of available beds in the local long-term units and significant staff reduction are limits to stop new admissions or implement a staff assignment. During this outbreak, patients could not be transferred into single bed-rooms, given the lack of available rooms and the psychological impact of confinement. The other infection control measures listed in methods section, were sufficient to contain and end the outbreak, suggesting cohorting measures might be adapted to patients’ specificities.

This study has certain limitations. Retrospective data collection could have led to bias and some clinical or epidemiological data are missing. Furthermore, sequencing of about half the samples failed, most likely due to low viral load. Notably, the RSV case from the SPR-C ward close to SPR-R one, couldn’t be linked to the outbreak. Finally, health care workers part of the transmission chain is unclear as information regarding symptoms, laboratory confirmation and adherence to control measures are lacking.

## Conclusion

In conclusion, we document here the relevance of virus genotyping for monitoring the spread of RSV during the epidemic, identifying the first case and discriminating between the epidemic cases and the concomitant sporadic cases. These data encourage the development of routine methods based on new high throughput sequencing methods that would allow these studies to be carried out in real time. Knowledge generated by these approaches improves understanding of viral emergence and spread and should contribute to better disease control.

## Supplementary Information

**Additional file 1: Sup. Figure S1.** Sociodemographic and clinical characteristics of patients from SPR RSV outbreak. a Defined by serum albumin concentration below 35 mg/L according to the biological definition by French health authority [23]. b Single oral temperature over 37,8 °C or repeated temperature over 37,2 °C. c Rhino pharyngitis (runny nose, sneezing or stuffy nose) or pharyngitis (sore throat, difficulty in swallowing). d Fever, chills and myalgia. e Defined by oxygen saturation level below 96%. f Chronic heart failure decompensation. g Seven days antibiotherapy.

## Data Availability

The datasets generated and/or analysed during the current study are available in GenBank under accession numbers MT989450 to MT98945062. The datasets used and/or analysed during the current study are also available from the corresponding author on reasonable request.

## Abbreviations

APHP: Assistance Publique des Hôpitaux de Paris
APR: Ambroise Paré academic hospital
CHF: Chronic Heart Failure
FEV_1_/FVC: Forced Expiratory Volume in 1 s / Forced Vital Capacity
GHUPIFO: “Paris Ile-de-France Ouest” hospital group
hMPV: Human Pneumovirus
LTCF: Long-Term Care Facilities
PIV: Parainfluenza Virus
RSV: Respiratory Syncytial Virus
SPR-CLR: Sainte-Perine-Chardon-Lagache-Rossini hospital
URTI: Upper Respiratory Tract Infection
LRTI: Lower Respiratory Tract Infection
CRP: C Reactive Protein
