# Supplementary figures and images for: Molecular investigation of a RSV outbreak in a geriatric hospital
Source: BMC Geriatr. 2021 Feb 12;21:120. doi: 10.1186/s12877-021-02064-6 (PMC7880219; doi:10.1186/s12877-021-02064-6)

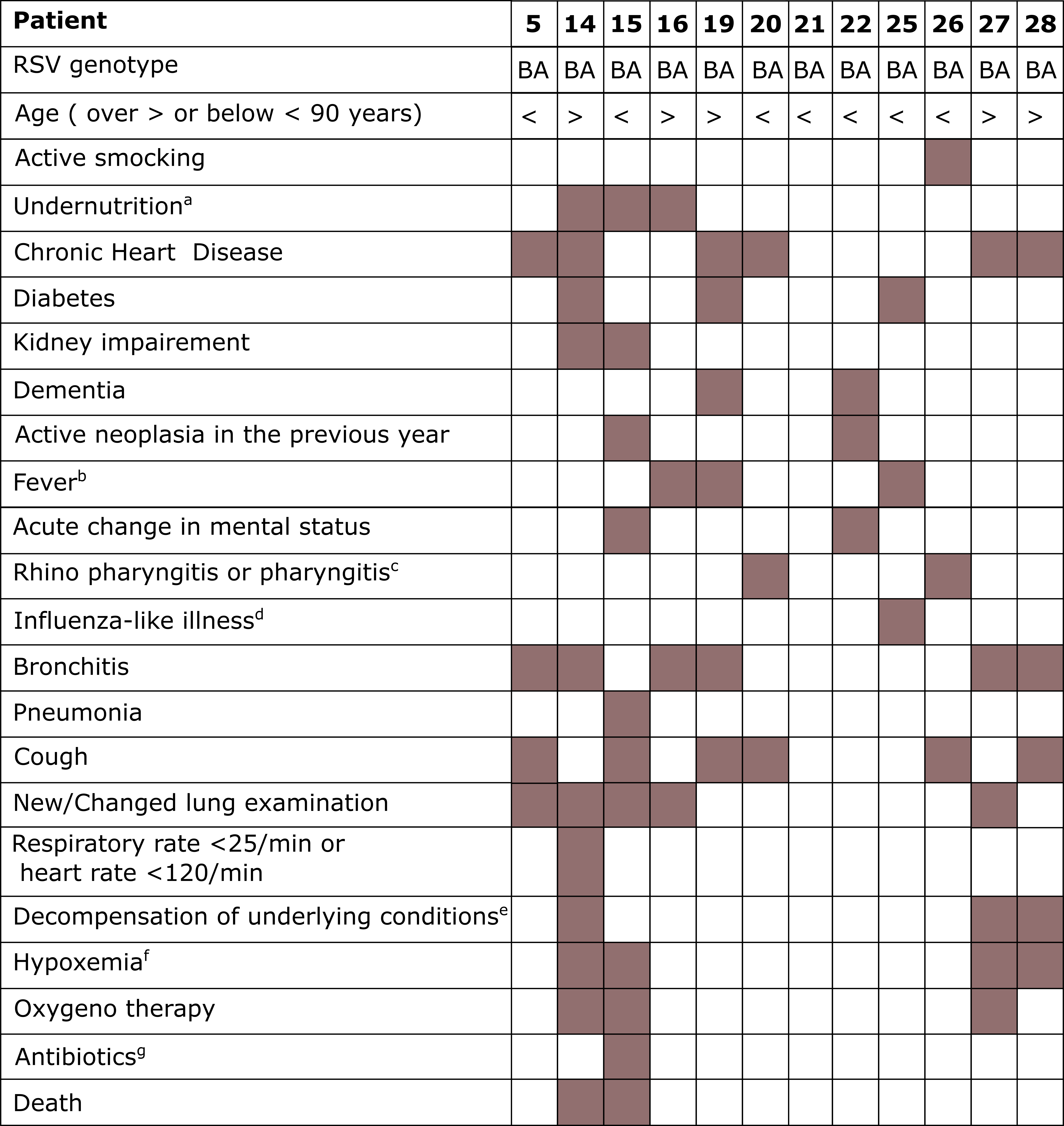

Supplement: Supplementary file 1 — Additional file 1: Sup. Figure S1. Sociodemographic and clinical characteristics of patients from SPR RSV outbreak. a Defined by serum albumin concentration below 35 mg/L according to the biological definition by French health authority [23]. b Single oral temperature over 37,8 °C or repeated temperature over 37,2 °C. c Rhino pharyngitis (runny nose, sneezing or stuffy nose) or pharyngitis (sore throat, difficulty in swallowing). d Fever, chills and myalgia. e Defined by oxygen saturation level below 96%. f Chronic heart failure decompensation. g Seven days antibiotherapy. [file 12877_2021_2064_MOESM1_ESM.png]
